# Partial loss of genes might open therapeutic window

**DOI:** 10.7554/eLife.25996

**Published:** 2017-03-17

**Authors:** Bo Liu, Omar Abdel-Wahab

**Affiliations:** 1 Human Oncology and Pathogenesis Program, Department of Medicine, Memorial Sloan Kettering Cancer Center, New York, United States; 2 Human Oncology and Pathogenesis Program and the Leukemia Service, Department of Medicine, Memorial Sloan Kettering Cancer Center, New York, United Statesabdelwao@mskcc.org

**Keywords:** copy number alterations, spliceosome, SF3B1, target identification and validation, CYCLOPS genes, cancer therapeutics, Human, Mouse

## Abstract

The loss of genes that encode RNA splicing factors weakens cancer cells in a way that could be exploited by new approaches to treatment.

**Related research article** Paolella BR, Gibson WJ, Urbanski LM, Alberta JA, Zack TI, Bandopadhayay P, Nichols CA, Agarwalla PK, Brown MS, Lamothe R, Yu Y, Choi PS, Obeng EA, Heckl D, Wei G, Wang B, Tsherniak A, Vazquez F, Weir BA, Root DE, Cowley GS, Buhrlage SJ, Stiles CD, Ebert BL, Hahn WC, Reed R, Beroukhim R. 2017. Copy-number and gene dependency analysis reveals partial copy loss of wild-type SF3B1 as a novel cancer vulnerability. *eLife* **6**:e23268. doi: 10.7554/eLife.23268

Copy number loss – which involves part of a chromosome being deleted during DNA replication – is a common occurrence in cancer. While the loss of tumor suppressor genes during copy number loss can promote the formation of tumors, the loss of other genes in these same regions was not thought to be relevant to cancer. In 2012, however, Rameen Beroukhim, William Hahn and co-workers analyzed a panel of 86 cancer cell lines to search for genes with a particular property – the deletion of one copy of the gene results in cells that are less able to withstand further down-regulation of gene expression than cells with two copies of the gene (Nijhawan et al., 2012). A total of 56 CYCLOPS genes were identified, many of which were found to encode proteins that are essential for cell survival. In general, genes that encode essential proteins are not pursued as drug targets, but the vulnerability of cancer cells that have lost one copy of an essential gene might create a therapeutic window.

Now, in eLife, Beroukhim, Robin Reed and co-workers – including Brenton Paolella and William Gibson as joint first authors – report how an analysis of 179 cell lines has allowed them to identify a total of 124 CYCLOPS candidate genes (Paolella et al., 2017). Many of these genes encode proteins that are part of a complex molecular machine called the spliceosome: in particular, the gene *SF3B1* encodes a protein that is the largest subunit in the SF3b complex which, in turn is part of a small nuclear ribonucleoprotein called the U2 snRNP complex. The spliceosome contains a total of five such ribonucleoproteins.

Although mutations in *SF3B1* have been observed in patients with a wide range of cancers, the loss of one copy of the gene is far more common than mutation. Across some 10,570 cases in the Cancer Genome Atlas, Paolella et al. – who are based at the Dana-Farber Cancer Institute, Harvard Medical School, Massachusetts General Hospital, Brigham and Women's Hospital and the Broad Institute – found that partial deletion of *SF3B1* occurs most frequently in chromophobe kidney cancer, urothelial bladder cancer, and breast cancer. The fact that none of the patients in the atlas had lost both copies of *SF3B1* is consistent with it being an essential gene. Previous work on *SF3B1* has shown that mutations and partial loss have distinct effects on splicing (Alsafadi et al., 2016).

Paolella et al. then performed a series of biochemical studies to explore how the partial loss of *SF3B1* effected the abundance of the SF3b and U2 snRNP complexes in cells that had lost a copy of the gene and cells that were copy neutral (that is, had not lost or gained a copy of the gene). These studies revealed that cells that had lost a copy of the gene were highly sensitive to further down-regulation of the gene, because they did not have sufficient levels of the SF3b complex, whereas the copy neutral cells were more tolerant to down-regulation of the gene (Figure 1).

**Figure 1.**
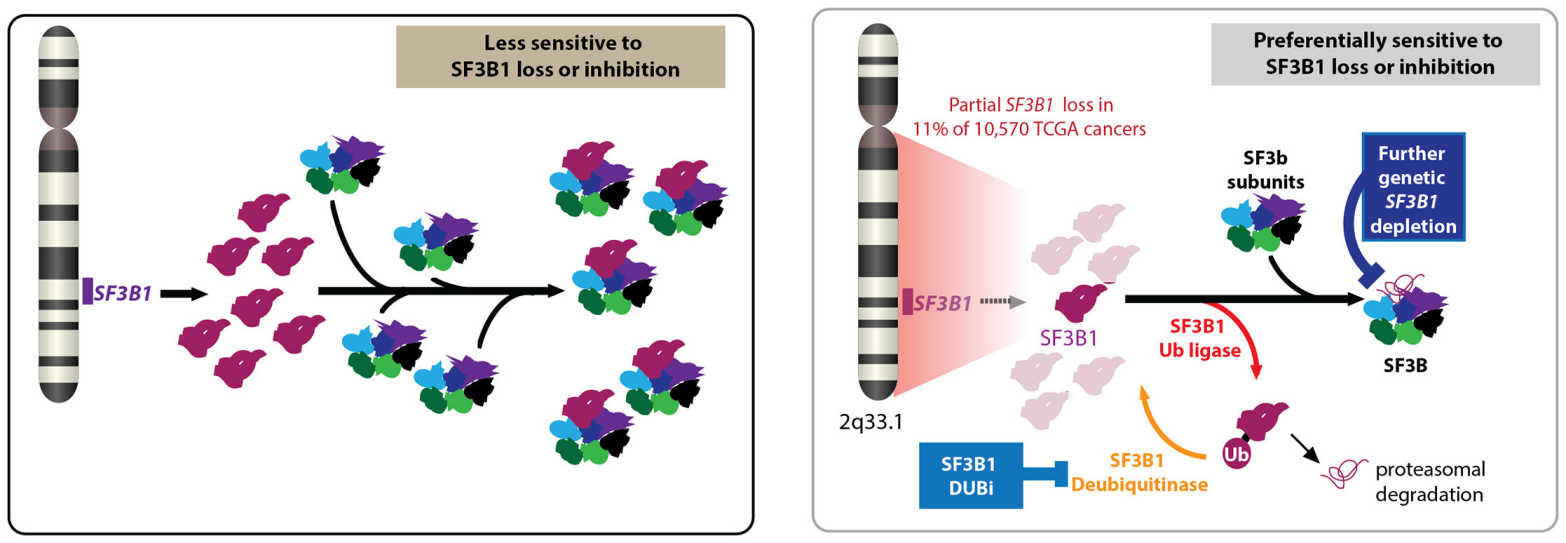
Partial loss of *SF3B1* and cancer. Left: The gene *SF3B1* encodes a protein (maroon) that is part of the SF3b complex (multiple colors), which is part of the spliceosome (not shown). Right: A significant number of cancer cells contain just one copy of *SF3B1*, rather than two, which leads to a reduced abundance of the SF3b complex in cells. Such cells are sensitive to further loss of *SF3B1* through genetic down-regulation (top) or ubiquitination by a ligase (bottom). Cancer cells employ deubiquitinases to reverse the effects of ubiquitination, so drugs that inhibit these deubiquitinases could be used to treat some cancers. CYCLOPS: copy-number alterations yielding cancer liabilities owing to partial loss; DUBi: deubiquitinase inhibitor.

In the past decade it has been shown that compounds that inhibit SF3B1 have potent anti-tumor activity (Kaida et al., 2007; Folco et al., 2011). These inhibitors appear to perturb U2 snRNP function without altering the level of the SF3B1 or U2 snRNP complexes. *SF3B1* mutant cells are more sensitive to these inhibitors than their wild-type counterparts (Obeng et al., 2016). However, partial loss of *SF3B1* does not appear to make cells more sensitive to these inhibitors.

In an effort to find a way to exploit the vulnerability of cells that had experienced a partial loss of *SF3B1*, Paolella et al. found that the regulatory protein ubiquitin had a central role in the function of the SF3B1 protein and in SF3B1-related cancer vulnerability (Figure 1). In particular, enzymes called ligases catalyzed the addition of ubiquitin to the SF3B1 protein, thus "tagging" it for degradation in the proteasome. However, other enzymes called deubiquitinases reversed this process, thus helping the cell to survive. Paolella et al. then showed that a drug called b-AP15, which was known to inhibit various deubiquitinases (D'Arcy et al., 2011), could inhibit the deubiquitinases in cancer cells that had experienced partial loss of *SF3B1*, and thus selectively kill these cells by reducing the amount of SF3B1 protein.

The next challenge is to identify the specific ubiquitin ligases and deubiquitinases for the SF3B1 protein, and to check for possible side effects of any drugs used to inhibit the relevant deubiquitinases.

Nonetheless, the work of Paolella et al. will, we hope, ultimately provide a number of new avenues for targeting the spliceosome in order to treat cancer.
